# Treating a Diseased Antrum

**Published:** 1892-05

**Authors:** 


ARTICLE III.

### TREATING A DISEASED ANTRUM.

Discussion in the Chicago Dental Society.

Dr. T. W. Brophy urges the necessity of thorough antiseptic cleanliness from the beginning to the end, without which it is almost impossible to cure disease of the antrum. We often find bone septum in the antrum separating the cavity into various cavities. It is sometimes difficult to make a diagnosis and to treat these cases well without breaking down the bone septum which separates the cavity into different parts. For instance, an operator may diagnose, as he thinks, disease of the antrum either by the presence of serous fluid or pus in the antral cavity. He may open the cavity and find it apparently in a healthy condition, and yet everything indicates he has an abscess. Further exploration will develop this anatomical peculiarity which is overlooked, the presence of the bone septum separating the cavity into two or three parts. I have seen cases where there were as many as eight distinct cavities formed, where there were little cavities running out into the molar process, distinctly formed, and separated from each other, so that, if fluid formed in one, we might make several explorations before coming in contact with it.

Drainage tubes are not generally necessary. If a large opening be made, which is the proper thing to do, making it as large as possible so as to introduce even the end of the finger, such an operation would give good drainage generally. The drainage tube must be just right, it must be neither too long nor too short. How can we determine the proper length ? If an operator takes a silver probe and bends its end so as to form a right angle, then introduce it into the antrum, he may hook it on the floor of the antrum, and by that means the distance between the floor of the antrum and the masticating surfaces of the teeth, thus indicating the length of the tube. He may then take platina and make a tube the right length. He should make a band in the manner we would proceed to make a band for a crown, and fit it to the tooth in close proximity to the opening or antral tube.

He can then, with molding compound, take an impression of the tube, and the band around the tooth, being careful to hold the tube where it ought to be. In taking that impression he removes the tube and also the band from the tooth, and makes a plaster cast surrounding them. He takes the cast to the laboratory, puts platina between the tube and band, and solders the two together, after which he adjusts the tube to the antrum, cementing the band to the tooth. He can drain the cavity at the most dependent part, which is at its floor. He has thus continuous drainage, which is the essential feature of the treatment of antral disease. When the patient is taking his food, he may plug the end with a little stopper, and thus prevent the introduction of food into the antrum. After meals he takes out the plug and gets continuous drainage. It seems to me this opening might be plugged with gauze, wax or anything else. Wax is clean, and I would not hesitate to use it. It will not absorb anything. It is often convenient about the mouth where I want to secure a wide opening, and to aid nature in filling in the cavity at its base with granulations; as, for instance, where we have necrosis, or carious bone. After the removal of the diseased bone, the wax may be introduced when the cavity has been antiseptically cleansed, and, by shaving off a little of the wax, from time to time, the granulations will fill the cavity and the disease be cured.

Irrigation is essential; and after this, I think one of the most valuable means of successful treatment is to make use of insufflations of powders, and there is nothing that serves me better than powdered boracic acid. This is my favorite remedy in these cases. An application that is especially desirable in all chronic inflammations of the mucous surface, attended with a low form of vitality and suppuration, is a solution of nitrate of silver in about one part to five thousand, enough to stimulate and bring about a healthy condition of the parts. Here we have a surface that is secreting pus. We first open the antrum and get rid of as much pus as we can by draining it, then we wash the cavity with some warm carbolized water, or any other weak solution, but do not use peroxide of hydrogen at first.

I saw a case, a day or two ago, where peroxide of hydrogen was injected in such a cavity as we are speaking about. The patient called on one of our practitioners, and he found what he supposed was a chronic abscess where a tooth had been extracted. He filled a rubber syringe with peroxide of hydrogen and carried it up into the socket of a bicuspid tooth, and let the fluid go. The patient told me that he thought he was going to lose his head. I said, "What do you mean?" "I really thought my head would burst." The peroxide of hydrogen entered the antral cavity which was half filled with pus, and you know what the result would be in such a case. The dentist did not observe the precaution of thoroughly irrigating the cavity with carbolized water or even warm water. He should have cleaned out the greater quantity of the pus, and then he could have made use of the peroxide of hydrogen and removed the little remnants on the mucous wall which the carbolized water would not remove. He would then have the cavity in shape to use boracic acid or whatever he wished. I would put boracic acid crystals in there and let them lie so as to get the prolonged action of the antiseptic. The crystals would dissolve slowly and would serve our purposes better than any fluid.

Usually, if we have teeth in these cases, I would make an opening large enough to secure drainage without a tube. I had an instance a little while ago in which there were no teeth; the gentleman who was treating the case made a tube and put it in a rubber plate, and that was a perpetual inconvenience to the patient, the movement of the plate, which always occurs to some extent, kept by irritation of the surrounding tissues. I would do it in this way: I take a large bur—I have some made for the purpose about four or five times as large as the largest burs used for excavating the cavities in teeth—and make an opening as large as my finger. By this means I secure continuous drainage, and the antrum is soon cured. The tube fixed to the plate is objectionable, because it causes more irritation than the patient can endure. A patient may wear a plate without much trouble, and the secretions will find their way down and out. I do not think it is advisable ever to put a tube in a plate where the teeth are all out, as tubes thus attached are certain to produce much inflammation of the surrounding tissues.

Dr. Louis Ottofy.—If the fluid you have injected comes out from the opening through the nostrils, would that be a sufficient indication that the antrum has been injected?

Dr. Brophy.—Generally it would. The opening between the antrum and the nose is situated almost at the summit of the antrum near the orbital plate. If you succeed in carrying the fluid up gently, you can irrigate the antral cavity.

I use a large syringe for this purpose. The syringe I use holds about four ounces of fluid. One of the important points in irrigation of the antrum is to use a large quantity of fluid, keeping the stream constantly running and thus washing the cavity. If you keep the stream flowing gently, it irrigates the cavity thoroughly and rids it of the fluids that may be there. I have a friend who endeavors to treat these antral diseases, where the teeth are present, by breaking down the naso-antral wall. I think this practice is objectionable. I have seen instances where there were thick incrustations of the nasal mucus in the antrum, and the incrustations formed there will keep up the inflammation and rather aggravate the condition. I would rather make the operation of Christopher Heath, which is also objectionable, that is to make an opening through the canine fossa. If we take a barrel, filled with fluid, put it on its end, and then take the plug out of the bunghole, it will drain the barrels down as far as the bunghole, but will not empty it. That is what occurs when we open the antrum at any other place than its base. Ninety per cent. of the cases of antral diseases are due to abscesses of the teeth, and, if we succeed in removing the pus which finds its way into the antrum from alveolar abscesses and keeping the cavity antiseptically clean, we can speedily cure the antral disease. The pus from alveolar abscesses, making its way into the antrum, establishes inflammation of the mucous membrane of the antrum; consequently disease of the antrum is a secondary condition. First we have abscess with all the preceding pathological changes, and then the antrum becomes involved to an extent which may lead to extensive caries, or necrosis of the bones which form the antral wall. Should the bones become diseased, treatment for the removal of the dead tissue must be employed.

I have been a close observer of the treatment of a friend of mine, who has a great many cases of diseases of the nose and air passages, and it seems to me that the mucus from the Schneiderian membrane accumulates in the cavity, and the method is, in my opinion, objectionable, because of the incrustations formed therein. I do not see how they can acccomplish as perfect drainage by the means outlined as by the method I have mentioned—opening at the base. Let us consider for a moment the structure of the antrum. For instance, we have at about the position of the first molar the most dependent part. I am not an artist, consequently I cannot make a drawing which would illustrate what I desire. The cavity is in the form of a pyramid, with the base toward the nose and the apex toward the molar process, and then dropping down so as to form something of a V-shape with the sharp angle of the V downward. You get that usually about the position of the first molar, and in cases where the teeth are responsible for the trouble,and owing to the fact that the first molars are the first to decay largely, in the greater per cent. of cases the molar is responsible for the antral disease, and therefore it is the one which we would naturally select to remove to secure the best drainage of the antral cavity. By removing the tooth, increasing the size of the opening of the roots, we can introduce a large tube and secure good drainage. I regard it as impossible to secure efficient drainage at the nasal orifice, or Schneiderian membrane. I regard the opening of the aveolar processes far more efficient than the nasal opening; besides, it gives the operator easier access to the antrum to treat it. It seems the use of warm air, after irrigating the cavity, would be advantageous before making use of powders. It has been my custom, where I am in doubt, to put the patient at night on the affected side, then direct him, if possible, to keep that position till morning, and suddenly turn to the healthy side. If the naso-antral opening be not closed, the fluid will be evacuated into the nasal passages, and it will pass into the pharynx when the fluid escapes, unless the patient leans his head forward. I can generally make a diagnosis by getting the patient to follow directions without the use of an exploring needle.

Dr. G. V. Clack.—I have listened to this with interest all the way. I have had considerable observation and experience in diseases of the antrum. Antral troubles are of three parts: First, the most frequent are those derived from tooth troubles; second, engorgements that come from irritations of the mucous membrane from cold, and various causes; third, tumors, or diseases that happen to be located in this region, but not growing on account of the anatomical forms here. I have seen all of these varieties, and, of course, they will assume great variety of form. The last case I examined proved to be epithelioma, not originating within the antrum, however, but extending to the antrum. It was a rather singular case. It seems to have originated on the cheek and had penetrated the masseter muscle, causing ankylosis of the jaw; and it was on this account that I was called to see the case. There was sufficient contraction of the muscles to close the mouth permanently. I found a large opening in the antrum in about the position of the wisdom tooth.

I have a patient now with a tube in the antrum. I put that tube in for the purpose of closing the opening, not to keep it open. The granulations which occur about the end of the tube that do not pass fully through, will affect the closure. I do not want continuous, but rather periodical drainage in these cases. I want it closed all the time, except when it is under my immediate observation. If it is to be opened at all, I want to be there. I do not want saliva to enter the cavity at all. The mucous membrane of the antrum is always affected if we allow saliva to pass in. It is infected through the nasal opening sometimes, but less extensively and injuriously than by the saliva. It is possible to keep the mucous membrane of the antrum free from suppuration for any length of time, provided we do not allow saliva to enter. Irritation by the saliva constitutes a very radical objection to the opening from the mouth in the treatment of the antrum, and, if I could find another point otherwise as good, I should prefer to use it and avoid opening from the mouth. An opening from the nasal passage is better in some respects, though we, as dentists, might object to it. The mucous from the nostrils might give some trouble. It is perfectly justifiable for those who are skilled in the treatment of the nasal cavity to use this kind of an opening. During the day there would not be perfect drainage. I would not have continuous drainage, and it is easy to get drainage when you handle cases with this kind of an opening. When I introduce a tube I plug it, and open it only when I am handling the case. I find little difficulty in the treatment of the antrum. I have had one or two, it is true, that did not do well; that is, pus would recur though they might seem well for a considerable time. But these have been rare.

I have one on hand where I removed a polypus from the antrum, which seems to have grown from a little spicula of necrosed bone, left after the removal of a tooth. To remove the polypus, it was necessary to make a large opening, so that I could introduce my finger into the antrum, and there is trouble in getting it to close. I do not like the idea of making a large opening, for I have several times had difficulty in getting it to close. We are sometimes obliged to make a plastic operation for its closure.

In this last mentioned, in which I removed a mass of semi-soft material, filling the antrum and producing a great deal of pressure, there was never any pus after the operation, and never any foul smell. After the removal of the growth or diseased part, I irrigated the antral cavity with a weak emulsion of the oil of cassia in warm water. It is not necessary really to have more than a solution to effect thorough disinfection. I use a single tube with a large bulb as a syringe, often using three or four bulbfuls in an irrigation. Then, after draining, close the opening from the mouth. If you have not a tube in place, you can use a wax plug. This medication acts kindly on mucous membranes, much more so than it does on the skin. It is not so liable to blister the mucous membrane as it is to blister the skin. If the oil of cassia is used too vigorously, it is an irritant; but not so much so as some of the articles mentioned, and it seems to me it is a much more effective antiseptic than anything else we have used in such positions. It is much more effective in such positions than bichloride of mercury. Some of our German friends have condemned the oil of cassia without giving it anything like a fair trial in actual work.

Dr. J. G. Reid.—I do not readily understand how saliva can enter the antrum on account of its situation, unless by capillary attraction.

Dr. Black.—When you have a hole into the antrum from the mouth, the saliva will enter through it. A patient may take water into his mouth and force it through the antrum and nostrils. Saliva will be forced in during the motions of mouth and tongue.

Dr. Brophy.—Mr. President, may I add a word to what I have said on the subject of drainage tubes? These fellows, called microbes, do not all swim, some of them fly. They are everywhere present. While I recognize the fact that saliva is one of the most active ferments that we have, I also recognize the fact that if we have continuous drainage, though the saliva should go into the antrum, which is doubtful, clinical history of these cases teaches us that they will soon get well if properly drained and antiseptically cleansed. The mouth is rarely, if ever, filled with saliva, the condition is different from filling the mouth with water, and using it as a pump to carry water into the antrum. The patient does not fill his mouth with saliva and then pump it into the antrum. If the saliva enters the antrum and we have continuous drainage, it would not do any harm. Ulcers of the mouth heal under the saliva; they heal under the tongue where saliva is always present. I am not prepared to accept the statement that the antrum must be kept free from saliva in order to get well. I am not prepared to say that we are to exclude it to hasten the cure. I have taken out the whole floor of the antrum on many occasions for the removal of tumors and diseased bone, and the saliva has not poisoned the freshly exposed surface, nor in any way retarded the process of repair. Only a week or two ago I opened both sides of the antrum, and there was very little inflammation. It healed up. The spaces which are sometimes open are made by the removal of necrosed bone, and they heal up without trouble in the presence of saliva. If you get an opening large enough, and get drainage, the saliva does no harm.

Dr. G. V. Black.—I am aware that the mucous membrane heals under the saliva. I have removed the floor of the antrum and found no difficulty in the healing process, and you will not where there is a continual washing of the parts with the saliva; but it is where the saliva is cooped up so that it will lodge and decompose that you have trouble, unless you keep the parts clean with a good antiseptic.

Dr. Brophy.—The saliva is not cooped up in the antrum, because you have constant drainage.

Dr. Black.—You have a little drainage tube to drain the part, but the hole is small and the antrum is large, you will have fermentation taking place in the antrum. When you put saliva into the cavity of a pulpless tooth what do you have? He who treats abscessed teeth without keeping saliva out knows he will get suppuration.

Dr. Brophy.—When you use drainage you get a free flow of saliva.

Dr. Black.—You will find that it is where the saliva does not flow freely that decomposition occurs, and there is where you get the trouble. The partial septi and irregularities of the antrum which prevent free flow, have been sufficiently described. I see these cases suppurating month after month—suppurating every day. The saliva and other foreign substances do not belong here, and they cause inflammation in such places much more certainly than ordinary mucus from the nostrils. We know saliva injected into rabbits is poisonous, and generally kills them. We know when we put human saliva in the tissues or in the cavities where it does not belong we get into trouble. At the Chicago College of Dental Surgery the students cut their fingers and applied court-plaster, and had suppurating fingers. If they had put a little cassia water on the plaster they would have avoided that, but they licked the paster. They poisoned themselves by their own saliva. At one time a great many of these cases occurred in that school, and the suppuration ceased when they used an antiseptic to wet their plaster.

Dr. J. G. Reid.—I would like to ask Dr. Black a question in regard to saliva. For instance, you have a dog, he cuts himself or is injured in some way. What does he do? He will lay down and lick that sore from morning till night till it gets well.

Dr. Black.—Yes, he keeps licking at it all the time and by so doing prevents fermentation taking place. Suppuration is the breaking down of inflammatory products by the process of fermentation.

Dr. Brophy.—We have to take into consideration the clinical history of these diseases. A patient comes to us with antral disease which he needs to have attended to. We know the first thing is to get the cavity drained. After we have secured drainage, emptied the cavity of its fermenting contents, we irrigate it, and apply our medicaments. My position on this subject is just this: If you plug a cavity, as one or two of the gentlemen have advocated to-night, so as to be sure that it is closed, you generally find that when it is opened you have considerable quantity of pus escaping at that time, and that pus, I contend, retained within the cavity is a great detriment to the tissues. Pus retards the healing of the tissues more than the saliva which might possibly get in the antrum, and I do not believe that saliva is likely to get in the antrum, except the patient makes a special effort to get it there. For that reason, I have followed both methods of treatment, viz.: keeping it plugged and keeping it open, and I have found in following up the clinical history of the cases, we get better results, a more speedy cure, if we keep the cavity open so as to drain it continuously. We cannot get constant drainage for the reasons that have been stated. The patient is not upright more than two-thirds of his time; the other third he is asleep. The antrum would certainly get well sooner by having it open at the floor. Plugging of the cavity will hold the secretions which are really irritating the tissues. I consider it far better to keep it open, even if a little saliva does get into it. Saliva on the mucous membrane produces less irritation than pent-up or retained or decomposing pus. The antrum should be irrigated two or three times a day to get a speedy cure. If it is plugged, you should remove the plug three or four times a day. If you do not do this, the patients go over Sundays and holidays and cannot get to the operator to have it treated. It is better to remove the pus than to have it in the antrum. The microbes are present there all the time from beginning to the end of treatment, and they will be there afterward. We have to keep in mind the fact that pus is forming and accumulating, and we must prevent it from remaining in contact with the diseased tissues, to restore the parts to health.—*Review.*

